# Mitochondrial Dysfunction and Metabolic Disturbances Induced by Viral Infections

**DOI:** 10.3390/cells13211789

**Published:** 2024-10-29

**Authors:** Sandra E. Pérez, Monika Gooz, Eduardo N. Maldonado

**Affiliations:** 1 Centro de Investigación Veterinaria de Tandil (CIVETAN), UNCPBA-CICPBA-CONICET, Campus Universitario, Tandil CC7000, Buenos Aires, Argentina; seperez@vet.unicen.edu.ar; 2 Department of Drug Discovery & Biomedical Sciences, Medical University of South Carolina, DD 506 Drug Discovery Building, 70 President Street, MSC 139, Charleston, SC 29425, USA; beckm@musc.edu; 3 Hollings Cancer Center, Medical University of South Carolina, Charleston, SC 29425, USA

**Keywords:** electron transport chain, glucose, glutamine, fatty acids, innate immunity, metabolic reprogramming, mitochondria, reactive oxygen species, VDACs, virus, Warburg

## Abstract

Viruses are intracellular parasites that utilize organelles, signaling pathways, and the bioenergetics machinery of the cell to replicate the genome and synthesize proteins to build up new viral particles. Mitochondria are key to supporting the virus life cycle by sustaining energy production, metabolism, and synthesis of macromolecules. Mitochondria also contribute to the antiviral innate immune response. Here, we describe the different mechanisms involved in virus–mitochondria interactions. We analyze the effects of viral infections on the metabolism of glucose in the Warburg phenotype, glutamine, and fatty acids. We also describe how viruses directly regulate mitochondrial function through modulation of the activity of the electron transport chain, the generation of reactive oxygen species, the balance between fission and fusion, and the regulation of voltage-dependent anion channels. In addition, we discuss the evasion strategies used to avoid mitochondrial-associated mechanisms that inhibit viral replication. Overall, this review aims to provide a comprehensive view of how viruses modulate mitochondrial function to maintain their replicative capabilities.

## 1. Introduction

As obligatory intracellular parasites, viruses use cellular pathways and organelles to meet their requirements for protein synthesis and replication. The life cycle of a virus is highly dependent on the contribution of mitochondria to the bioenergetics, metabolic, and biosynthetic capabilities of cells. Mitochondria also induce antiviral innate immune mechanisms, like the activation of the mitochondrial antiviral-signaling protein (MAVS) [1] and the type I interferon (IFN) response via the release of mitochondrial DNA (mtDNA) [2]. Furthermore, mitochondria participate in antiviral-signaling pathways, including the activation of toll-like receptors (TLRs) [3] and the NLRP3 inflammasome [4].

**Figure 1:**
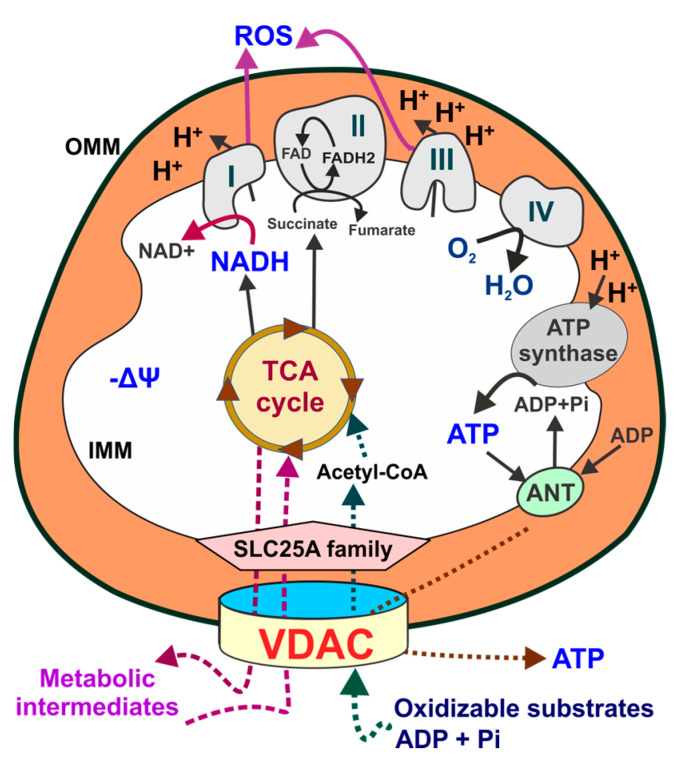
Schematics of mitochondrial metabolism. Oxidizable substrates, ADP and Pi, cross the outer mitochondrial membrane through VDACs. Acetyl-coenzyme A, generated from respiratory substrates, enters the TCA cycle, generating NADH and FADH_2_, which fuel the electron transport chain to support oxidative phosphorylation. The TCA cycle also produces metabolic intermediaries released to the cytosol for the synthesis of proteins and lipids. H^+^ pumping by the respiratory chain across the inner mitochondrial membrane generates a ΔΨ and a proton motive force used by the F1F0-ATP synthase (complex V) to synthesize ATP. Mitochondrial ATP is exported from the matrix by the ANT and released to the cytosol through VDACs. The flow of electrons through complexes I, II, and III also generates ROS. AcCoA: Acetyl CoA; ANT: adenine nucleotide transporter; α-KG: alpha-ketoglutarate; IMM: inner mitochondrial membrane; OMM: outer mitochondrial membrane; Pi: inorganic phosphate; ROS: reactive oxygen species; VDACs: voltage-dependent anion channels; ΔΨ: mitochondrial membrane potential.

Mitochondrial function and metabolism are supported by fully oxidizable substrates. The tricarboxylic acid (TCA) cycle (also known as Krebs cycle) in the mitochondrial matrix is fueled by the metabolic intermediate acetyl-coenzyme A, which is generated by the oxidation of glucose-derived pyruvate, β-oxidation of fatty acids (FAs), and the amino acids leucine, isoleucine, glycine, serine, and tryptophan. A major byproduct of the TCA cycle is NADH, which is a major donor of the electrons (e^−^) that flow through the complexes of the electron transport chain (ETC) to reduce the final acceptor molecular O_2_ to H_2_O. Oxygen consumption is the parameter used to measure cellular respiration. The flow of e^-^ through the ETC also produces two parallel phenomena: a proton (H^+^) translocation across the inner mitochondrial membrane and an e^-^ leak. Translocated H^+^ at complexes I, III, and IV accumulate in the mitochondrial intermembrane space, generating a negative transmembrane potential (mitochondrial membrane potential, ΔΨm) and a positive Δ*p*H, both components of the proton motive force (Δ*p*). Δ*p* is the force that drives ATP synthesis from ADP and inorganic phosphate (Pi) by Complex V (F_1_F_O_-ATP synthase). In non-cancerous and non-proliferating cells, mitochondria produce ~95–98% of total cellular ATP through oxidative phosphorylation (OxPhos). Thus, mitochondrial function is sustained by coupling substrate oxidation with ATP synthesis [5]. Beyond bioenergetics, an e^-^ leak at complexes I, II, and IV of the respiratory chain generates reactive oxygen species (ROS). Quantitatively, mitochondria are the major source of ROS. ROS generation in mitochondria is highly dynamic and modulated by changes in mitochondrial metabolism. Total cellular ROS depends on the balance between production and detoxification by enzymatic and non-enzymatic antioxidants, both in mitochondria and cytosol, that prevent excessive ROS accumulation [6]. Superoxide anion (O2•^−^), hydroxyl (OH•), peroxyl (ROO•) and alkoxy (RO•) radicals, and hydrogen peroxide (H_2_O_2_) originate from molecular O_2_ [7]. Superoxide dismutases are a family of scavenging enzymes that convert O2•^−^ to H_2_O_2_, which is then further reduced to H_2_O by catalase, glutathione peroxidases, and peroxiredoxins [3]. Only O_2_•^−^ and H_2_O_2_ act as signaling molecules that contribute to maintaining cellular homeostasis and proliferation [8]. The highly toxic HO• and peroxynitrite (ONOO^−^), for which there are not specific detoxifying systems, do not regulate biochemical pathways. Mitochondrial ROS are either released to the cytosol, mainly H_2_O_2_, as signaling molecules or converted to non-reactive species by antioxidant systems. If the antioxidant capability of the cells is exceeded, accumulated ROS cause oxidative stress [9] (Figure 1).

Mitochondria are also metabolic hubs that generate metabolites through the catabolism of pyruvate, glutamine, and other respiratory substrates in the TCA cycle. When glucose levels are sufficient for energy generation, α-ketoglutarate and oxalo-acetate, which are glutamine derivatives, are utilized for the synthesis of nonessential amino acids. Citrate, exported to the cytosol, is converted into acetyl-coenzyme A and utilized for the synthesis of FA, cholesterol, and amino acids. Furthermore, glutamine provides nitrogen for the synthesis of purine and pyrimidine and is a precursor for the synthesis of glutathione [6,10,11,12,13,14]. Recently, the transfer of one-carbon units from serine and glycine by a process known as one-carbon metabolism has been shown to be involved in the de novo synthesis of purines and thymidylate synthase in highly proliferative tumors [15].

Mitochondria continuously adapt to metabolic demands by changing the number and morphology through mitochondrial fission, fusion, biogenesis, and mitophagy. Recently, defective mitophagy has been linked to the risk of autoimmune disease due to the accumulation of mtDNA [2].

Because of the multiple roles in bioenergetics and metabolism, mitochondria are essential to maintain cellular homeostasis and a necessary target to favor viral replication. This review summarizes the mechanisms involved in the interactions between viruses and mitochondria. We also describe how viruses benefit from using mitochondrial metabolism and avoid the mechanisms associated with mitochondria that inhibit viral replication. A better understanding of these mechanisms may help to develop novel strategies to control viral infections.

## 2. Mitochondrial Reactive Oxygen Species during Viral Infections

### 2.1. Oxidative Stress and Beneficial Effects of Reactive Oxygen Species

ROS generated during viral infections can either favor viral replication and the progression of disease or be deleterious to the infected cell. The outcome depends on the amount of ROS accumulated. Oxidative stress occurs when the production exceeds the cellular antioxidant capacity and the ability to eliminate the reactive intermediates, resulting in damage to DNA, proteins, and lipids [7].

ROS-induced damage to macromolecules has been recognized as a key factor in the pathogenesis of diseases like viral encephalitis, which causes neuronal damage in the central nervous system (CNS) [16]. In the CNS, ROS are mainly produced by microglial cells in response to inflammation or tissue injury [17]. Viruses have evolved strategies to avoid antiviral responses or premature apoptosis caused by excessive accumulation of ROS [18]. *Dengue virus* (DENV) serotype 4, *Japanese encephalitis virus* (JEV), r*abies virus*, and *human immunodeficiency virus* (HIV) cause oxidative stress in the CNS. The detrimental effects of ROS on neurons after DENV-4 have been demonstrated by Suwanprinya et al. [19]. Although the exact mechanism of ROS production induced by DENV-4 has not been identified, virus attachment to a series of receptors like glycosphingolipids or a component of the viral particle might cause this phenomenon at the early phase of infection. Excessive ROS production, which is more evident after DENV infection progresses, is attributed to a decreased antioxidant response mediated by nuclear factor erythroid 2-related factor 2 (Nrf2) that is targeted by a DENV NS2B3 protease complex. The absence of this antioxidant regulation triggers an increase in ROS levels that favors virus replication, along with upregulation in the expression of inflammatory and apoptotic genes [20]. Consistent with this, an imbalance between ROS and the antioxidant systems has been linked to the development of severe disease [21], mainly mediated by the release of pro-inflammatory cytokines [22], leading to the vascular dysfunction characteristic of severe DENV infection [23]. Furthermore, DENV decreases intracellular GSH levels, which also promotes DENV replication [24]. After JEV infection of neurons, there is a significant upregulation of pyruvate dehydrogenase kinase 1 (PDK-1), stimulating the generation of free radicals. It has been suggested that PDK-1 phosphorylation and the inhibition of the pyruvate dehydrogenase (PDH) complex leads to the accumulation of ROS [25], which contributes to neuronal apoptosis [26]. Oxidative stress during *rabies virus* infection of dorsal root ganglia cultures contributes to neuronal degeneration. The interaction of the *rabies virus* P protein with complexes I and IV of the ETC resulted in increased respiration and ROS production [27,28]. Mitochondrial dysfunction was also associated with a high ΔΨm, high NADH/NAD+ ratio, and low levels of ATP [27]. *Human immunodeficiency virus* (HIV) invades the nervous system through the trafficking of infected immune cells. Infected CNS resident cells, mainly astrocytes, avoid apoptotic death triggered by virus-induced mitochondrial fragmentation. Clearance of injured mitochondria by mitophagy results in astrocytes’ survival. Consequently, infected surviving astrocytes become a long-term virus reservoir in the brain that can also induce bystander cell death caused by mitochondrial dysfunction induced by high levels of mitochondrial ROS [29]. This event relies on the HIV accessory proteins Nef, Vpu, and Vpr and involves mitochondrial membrane depolarization, with cell death triggered by caspase-dependent and independent mechanisms [30].

It has been shown that non-toxic levels of ROS enhance viral replication [18,31,32,33,34]. While several viral proteins involved in ROS formation have been studied, other potential mediators of viral ROS modulation are yet to be identified [31]. The beneficial effects of ROS on the life cycle of viruses, starting as early as a virion binds to its cell receptor, have been demonstrated using antioxidants both in vitro and in animal models [34]. Particularly, RNA viruses are prone to ROS-induced modifications [35]. Viral mutations and the immunosuppressive effect of ROS may contribute to the selection of more virulent strains that escape the immune response [35,36,37]. Viruses that benefit from ROS formation include *lymphocytic choriomeningitis virus* (LCMV), *Kaposi’s sarcoma-associated herpesvirus* (KSHV), *respiratory syncytial virus* (RSV), *influenza A virus* (IAV), and *hepatitis C virus* (HCV). Michalek et al. [38] demonstrated increased ROS levels 15 min after the attachment of LCMV to the cell surface. After a weak initial binding, increased ROS levels and receptor modifications induced by ROS, strengthened the virion/cell receptor interaction, favoring the progression of the infection. Furthermore, ROS sensitized the neighboring cells to LCMV binding. The initial phase of ROS generation after infection was followed by a second wave, which was required for efficient viral replication. Similarly, a very early induction of ROS production favored the binding of the KSHV to endothelial cells in the microvasculature [32]. The effects of RSV, which causes severe respiratory disease, on mitochondrial function have been widely studied [39,40,41]. Eighteen hours post-infection (hpi) of adenocarcinoma alveolar cells, increased mitochondrial ROS generation favored RSV replication. This effect was blocked by treatment with MitoQ, a mitochondrial antioxidant, which acted as a potent inhibitor of RSV infection. Furthermore, in a murine model, MitoQ treatment reduced RSV titers and lung inflammation, indicating the importance of mitochondrial ROS production in the pathogenesis of the disease [41]. IAV is another respiratory pathogen that benefits from ROS production in infected cells. IAV infections are responsible for annual, epidemic respiratory diseases, with some strains having pandemic potential [42]. Antioxidant treatment in IAV-infected mice with an intranasally delivered mitochondrial ROS scavenger reduced mortality and lung inflammation [33]. This was consistent with an increase in the type I IFN response and a reduction in IAV titers in the lungs, accompanied by a decrease in pro-inflammatory cytokines at the late stages of infection. Beneficial effects of ROS have also been described for HCV, which causes chronic and persistent infections that are frequently associated with chronic hepatitis, cirrhosis, and hepatocellular carcinoma [43]. Among several HCV proteins that regulate oxidative stress, the *core* protein—a structural protein that targets the outer mitochondrial membrane—is considered the most potent regulator [44,45]. ROS induction is mediated by the binding of HCV *core* protein and the inactivation of heat shock protein (Hsp60), a stress response molecular chaperone mainly localized in the mitochondrial matrix. Protein misfolding caused by Hsp60 inactivation leads to mitochondrial dysfunction and ROS production, which in turn sensitizes cells to apoptosis induced by tumor necrosis factor-alpha (TNF-α) [46].

**Figure 2:**
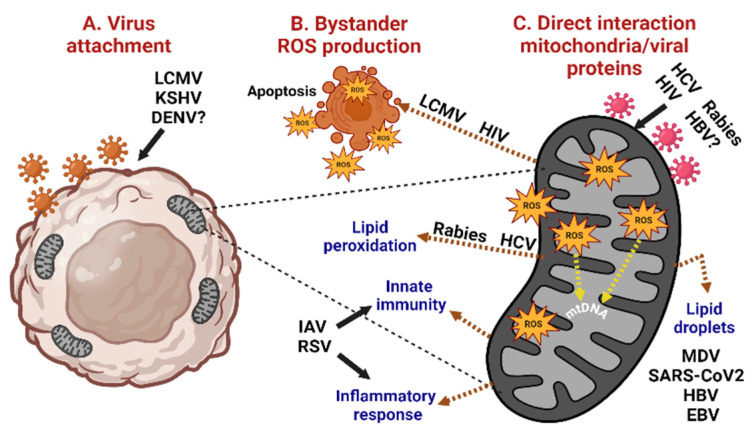
Mitochondrial ROS production and the effects on virus infection. (**A**) Virus attachment triggers ROS generation. (**B**) ROS favors further virus binding to neighboring cells (bystander effect), which may lead to apoptotic cell death. (**C**) Direct interaction of viral proteins with mitochondrial components induces ROS production, leading to apoptotic cell death, alterations in lipids metabolism, activation of innate immunity, and the inflammatory response. DENV: *dengue virus*; LCMV: *lymphocytic choriomeningitis virus;* HIV: *human immunodeficiency virus;* KSHV: *Kaposi’s sarcoma-associated herpesvirus;* HCV: *hepatitis C virus*; HBV: *hepatitis B virus*; MDV: *Marek´s disease virus;* EBV: *Epstein–Barr virus;* RSV*: respiratory syncytial virus;* IAV*: influenza A virus;* mtDNA: mitochondrial DNA.

As described above, some viruses trigger mitochondrial ROS production to favor virulence and viral replication, while others regulate ROS generation to induce cell death or foster cell survival in the case of persistent chronic infections (Figure 2).

**Figure 3:**
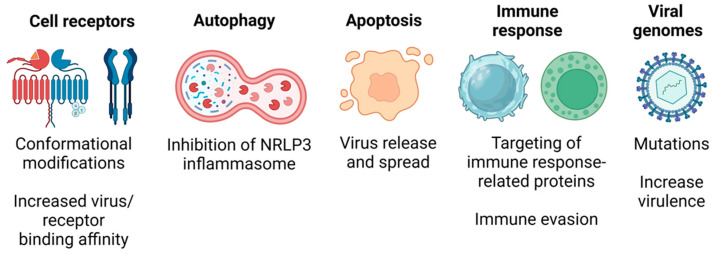
ROS-mediated mechanisms favoring viral infections. ROS-induced conformational changes on cell receptors favor virus adsorption, trigger autophagy that leads to inflammasome inactivation, stimulate apoptotic cell death to allow virus release and spread, alter viral proteins that favor evasion of the immune response, and introduce mutations to the virus genome, increasing virulence.

Overall, the beneficial effects of ROS include (a) changes in the conformation of cell receptors that increase virus/cell receptor binding affinity, (b) increased autophagy to repress inflammasome activation, (c) sensitization of cells to the intrinsic apoptotic pathway promoting virus release and spread, (d) targeting of proteins involved in the immune response favoring immune evasion mechanisms, and (e) direct ROS-mediated induction of mutations on the viral genomes increasing virulence (Figure 3).

### 2.2. Cell Death Mediated by Reactive Oxygen Species

Some viruses inhibit cell death to amplify their progeny or to persist in the host, while others promote cell death to spread out to other cells or tissues. Mitochondrial ROS are directly associated with different types of cell death, such as apoptosis, necroptosis, pyroptosis, and ferroptosis, all of which can occur during viral infections (Table 1).

The role and the mechanisms of mitochondrial ROS in virus-induced apoptosis have been well-characterized and reviewed previously [18,47,48]. *Sendai virus* [49], *SARS-CoV-2* [50], *porcine epidemic diarrhea virus* [51], *Rift Valley fever virus* [52], HIV-1, HCV, and IAV [47] are among the several viruses associated with ROS-induced apoptosis. The mechanisms of ROS in apoptotic cell death involve the activation of AMP-activated protein kinase (AMPK), which upregulates the transcription factor E2F1 and, consequently, the transcription of pro-apoptotic genes [53]. The intrinsic apoptotic pathway can also be induced by ROS-mediated Bax activation and insertion into the outer mitochondrial membrane, leading to cytochrome C release and activation of downstream caspases, such as the key apoptosis executioner, caspase 3. Additionally, caspases can cleave the p75 subunit of complex I in the ETC, which is followed by ROS generation and further amplification of the apoptotic signals [53,54].

Necroptosis is a type of regulated cell death with morphological characteristics that resemble necrosis. Engagement of cell surface death receptors, such as Fas and tumor necrosis factor-alpha receptor 1, cause loss of plasma membrane integrity, leading to the release of damage-associated molecular patterns (DAMPs) that promote an inflammatory response [55]. The execution of necroptosis relies on the receptor-interacting protein kinases 1 and 3 (RIPK1 and RIPK3) and the pseudokinase mixed-lineage kinase domain-like (MLKL) proteins. Increased mitochondrial ROS induces the mitochondrial translocation of p53 and RIPK1 phosphorylation. RIPK3 is recruited to the necrosome and phosphorylated by RIPK1. Then, RIPK3 phosphorylates MLKL, triggering its oligomerization and destabilizing the plasma membrane [53,55,56]. Furthermore, ROS has been implicated in disulfide bond formation between MLKL subunits, a prerequisite to induce necroptosis [57]. It has been shown that RSV infection of human macrophages induces TLR4 and TLR3 activation and ROS generation, which in turn trigger a RIPK1-independent, TRIF-dependent RIPK3-MLKL necroptotic pathway [58]. Increased ROS production in *Theiler’s murine encephalomyelitis virus* (TMEV) infection of macrophages has also been linked to necroptotic cell death, particularly when apoptosis is inhibited [59]. Necroptosis has been described for many viruses, including HIV-1 [60], *vaccinia virus* [61], MCMV [62], and reoviruses [63]. However, a direct link between necroptosis and ROS signaling has not been clearly established or investigated [64].

Pyroptosis is another regulated lytic type of cell death that requires ROS for activation of the NLRP3 inflammasome that triggers caspase-1 cleavage and activation of the pore-forming protein gasdermin, leading to membrane permeabilization and cell death [55]. Therefore, pro-inflammatory mediators (IL-1β and IL-18) are released [65]. RSV infection of macrophages activates TLR2 and ROS production, with ROS having a critical role as a second signal for inflammasome activation [58]. DENV infection or treatment of human endothelial cells with DENV recombinant E protein also induced pyroptosis, necroptosis, and ferroptosis. However, the highest level of cell death has been attributed to ROS-mediated pyroptosis and caspase-1 activity [66]. Similarly, high levels of ROS production and inflammasome activation were evident in the platelets from DENV-infected patients [67].

Ferroptosis is characterized by an iron overload, accumulation of ROS, lipid peroxidation, depletion of glutathione (GSH), and alterations of mitochondrial morphology [55,68]. Morphologically, ferroptotic cells show disruption of the plasma membrane, chromatin condensation, and mitochondrial and nuclear swelling [68]. The exact mechanism of ROS participation in ferroptosis has not been fully elucidated. Viruses may induce ferroptosis to replicate and evade the host immune system [69]. Ferroptotic cell death has been described in the HSV-1 infection of cultured neural cells and in a mouse model of encephalitis. Following HSV-1 infection, Nrf2, which regulates the expression of antioxidative genes, is ubiquitinated and degraded, thus disturbing cellular redox homeostasis and promoting ferroptosis [68]. Furthermore, ferroptosis is considered an effective mechanism for tumor suppression [70]. For example, the *Newcastle disease virus* (NDV) selectively induces ferroptosis in tumor cells initiated by the activation of p53, followed by nutrient deprivation, suppression of the cystine-glutamate antiporter system Xc^-^, and induction of ferritin degradation, which stimulates iron release [71]. Iron metabolism dysfunction in COVID-19 patients is associated with ferroptotic cell death in multiple organs. In this case, SARS-CoV-2 infection decreased the GSH pool and downregulated GPX4 gene expression [72]. Ectopic expression of *hepatitis A virus* (HAV) 3C protease (3Cpro) induced ferroptosis in several human cell lines. However, low cellular levels of 3Cpro did not induce cellular alterations compatible with ferroptosis. Therefore, it has been suggested ferroptosis is a side effect of 3Cpro activity in certain cell types [73]. Latent *Epstein–Barr virus* (EBV) has also been implicated in the induction of ferroptosis in newly infected human primary B lymphocytes and in transformed B cells. The levels of ferroptotic death vary with the levels of susceptibility according to the distinct states of latency, with Burkitt´s lymphoma cells being highly vulnerable to ROS-induced lipid peroxidation and, consequently, to ferroptosis [74]. Furthermore, these cells have low GSH stores and limited capacity for cystine uptake [75]. *Swine influenza virus* (SIV) infection increased the iron levels in adenocarcinoma alveolar cells and inhibited the GPx4/system Xc-axis, which is essential for avoiding lipid peroxidation. Thus, high concentrations of intracellular iron, combined with elevated levels of ROS and lipid peroxidation, ultimately caused the death of infected cells by ferroptosis [76].

**Table 1:** Types of cell death induced by ROS.

Cell Death Type	Features	General Mechanisms Involving ROS	Virus	Model	References
Necroptosis	✓ Resembles necrosis ✓ Disruption of plasma membrane ✓ Swelling of organelles ✓ Leakage of intracellular components	✓ Engagement of cell surface death receptors mROS-induced translocation of p53, phosphorylation of ✓ RIPK1-3 and MLKL ✓ mtROS-induced MLKL-disulfide bond formation	RSV	Human monocyte cell line (THP-1)	[58]
			TEMV	Murine macrophage culture	[59]
			DENV	Human endothelial cells (HMEC-1)	[66]
Pyroptosis	✓ Swelling and rupture of cell membranes ✓ Alteration of nuclear morphology ✓ Release of pro-inflammatory components	✓ Inflammasome activation ✓ Caspase-1 ✓ Gasdermin (membrane pore formation)	RSV	Human monocyte cell line (THP-1)	[58]
			DENV	Human endothelial cells (HMEC-1) and platelets	[59,67]
Ferroptosis	✓ Plasma membrane disruption ✓ Loss of mitochondrial potential, ✓ Partial chromatin condensation ✓ Nuclear and mitochondrial swelling	✓ Iron-dependent ROS accumulation. ✓ ROS-induced lipid peroxidation, ✓ GSH depletion ✓ Inactivation of GPX4 ✓ Mitochondrial morphological alterations	HSV-1	Neural cells Encephalitis murine model	[68]
			NDV	Glioma cells	[71]
			HAV	Ectopic HAV 3C protease expression in human cell lines	[73]
			EBV	Human primary B cells	[74]
			SARS-CoV2	Vero (African green monkey) cells	[72]
			SIV	Adenocarcinoma alveolar cells	[76]
			DENV	Human endothelial cells (HMEC-1)	[66]

mtROS: mitochondrial ROS; RIPK1-3: receptor-interacting protein kinases 1 and 3; MLKL: pseudokinase mixed-lineage kinase domain-like; GSH: glutathione; GPX4: glutathione peroxidase 4; 9RSV: *respiratory syncytial virus;* TEMV: *Theiler’s murine encephalitis virus;* DENV: *dengue virus*; HSV-1: *herpes simplex virus 1*; NDV: *Newcastle disease virus;* HAV: *hepatitis A virus;* EBV: *Epstein–Barr virus;* SIV: *swine influenza virus.*

## 3. Metabolic Effects of Viral Infections

**Figure 4:**
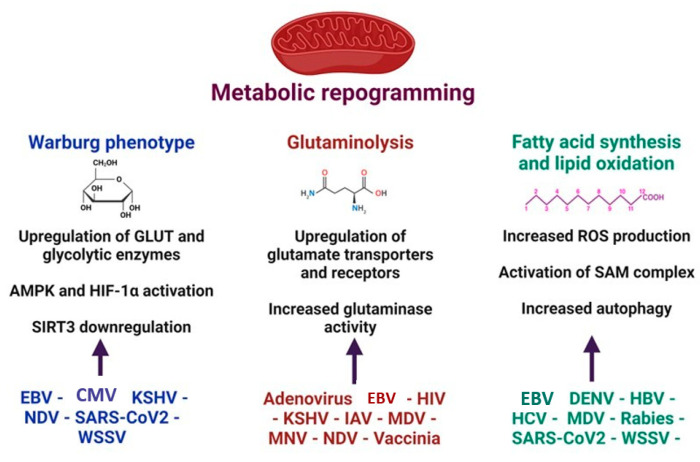
Major mitochondrial metabolic pathways and global mechanisms activated during viral infections. Virus replication is an energy demanding process. To cope with this energetic demand, viruses induce cellular metabolic reprogramming, which includes enhanced glycolysis (Warburg phenotype), glutaminolysis, fatty acids synthesis, and lipid oxidation. KSHV: *Kaposi´s sarcoma-associated herpesvirus;* HCMV: *human cytomegalovirus;* EBV*: Epstein–Barr virus;* NDV: *Newcastle disease virus;* MNV: *murine norovirus*; MDV: *Marek´s disease virus*; HBV: *hepatitis B virus*; HCV: *hepatitis C virus;* IAV*: influenza A virus;* WSSV*: white spot syndrome virus;* HIV*: human immunodeficiency virus.* GLUT*: glucose transporter;* AMPK: AMP-activated protein kinase; SAM complex: β-barrel-specific sorting and assembly machinery; mtROS: mitochondrial ROS.

Virus replication is energetically and metabolically very demanding [77]. Viruses cope with these demands, inducing metabolic reprogramming, including the upregulation of enzymes that control the metabolic pathways [78,79]. The metabolic switch enhances glycolysis (Warburg effect), glutaminolysis, and lipid oxidation [78] (Figure 4). An upregulated pentose phosphate pathway and changes in amino acid metabolism after viral infections have also been reported [80]. Some viruses may turn on more than one pathway to support their energetic needs, while different cellular metabolic pathways may be activated at distinct stages of the virus life cycle [81,82].

### 3.1. Warburg Phenotype

The Warburg phenotype, characterized by enhanced glycolysis, even in the presence of physiological concentrations of O_2_, is displayed by cancer cells and cells with high proliferation rates, like activated T lymphocytes. OxPhos generates 95–98% of the total cellular ATP in quiescent cells, whereas glycolysis and the succinyl-CoA ligase reaction in the TCA cycle provide the remaining 5%. By contrast, cancer cells generate ~10–90% of the total ATP by glycolysis [83,84,85]. Full oxidation of glucose in mitochondria generates ~32 moles of ATP, but only 2 moles of ATP/mole of glucose are produced during glycolysis [83]. Despite this difference in the yield of ATP and arguments in favor or against a higher rate of glycolysis being able to compensate for the relatively lower efficiency, the reality is that the ATP demand for cell division is low compared to the energy requirements for maintaining cellular functions, mainly the activity of the Na^+^-K^+^ ATPase. This fact strongly suggests that ATP generation is not limiting for rapid cell proliferation [86,87,88,89]. The current consensus is that enhanced aerobic glycolysis, together with mitochondrial metabolism, is necessary to provide not only ATP but also metabolic intermediates for the synthesis of macromolecules [90,91,92,93]. The shift towards aerobic glycolysis has also been proposed to increase resistance to apoptosis [94,95].

The mechanisms and changes in glucose metabolism induced by viruses are summarized in Table 2.

An increased expression of glucose transporters (GLUT) and enzymes involved in the glycolytic pathway has been shown in several viral infections [78]. *Human cytomegalovirus* (HCMV) replication increased glucose uptake [96] by downregulating GLUT1 and upregulating the more efficient GLUT4 [97]. HCMV infection also upregulated the glycolytic enzymes phosphofructose kinase-1, hexokinase, PDH, and also increased glycolytic intermediates and lactate [98,99,100,101]. In addition, these changes in glucose metabolism increased FA and lipid synthesis [101]. GLUT3 and hexokinase II have been found to be upregulated in KSHV-latently infected endothelial cells [94].

Several viral infections cause enhanced glycolysis and metabolic reprogramming through the activation of hypoxia-inducible factor 1-alpha (HIF-1α). An initial infection with SARS-CoV2 is characterized by enhanced aerobic glycolysis and high virus titers [82]. Elevated glucose levels promote viral replication and cytokine expression, leading to rapid tissue dissemination and exacerbated inflammation [82,102]. In monocytes, enhanced glycolysis and the pro-inflammatory state responsible for the lung injury observed in COVID-19 patients were mediated by HIF-1α [102]. *Epstein–Barr virus* (EBV) is a human oncogenic gamma-herpesvirus that is associated with several types of lymphocytic disorders and epithelial tumors [103]. The EBV latent membrane protein (LMP)-1 oncoprotein regulates tumorigenesis and shifts the metabolic program towards aerobic glycolysis by activating HIF-1α [104,105,106]. KSHV also activates HIF-1α, which is required for activating KSHV oncogenes [79].

In other cases, the Warburg effect in virus-infected cells is accompanied by decreased activity of the mitochondrial respiratory chain complexes and mitochondrial dysfunction without a complete shutdown of OxPhos. *Newcastle disease virus* (NDV) is an avian virus oncolytic to mammalian cells [107]. Recently, it was shown that NDV induces mitochondrial damage, leading to the degradation of SIRT3 via mitophagy [108]. SIRT3 is a mitochondrial member of the sirtuin family of the NAD-dependent ADP-ribosyl transferases and/or protein deacetylases involved in metabolism and stress response [109]. Reduced or lack of SIRT3 activity shifts mitochondrial bioenergetic metabolism toward glycolysis, which contributes to viral replication [108].

Finally, a mitochondrial metabolic switch has also been well-characterized in viral infections of crustaceans caused by the *white spot syndrome virus* (WSSV). The stage of viral genome replication in the shrimp immune cells is characterized by increased expression and activity of glycolytic enzymes, such as hexokinase and phosphofructokinase [110]. Furthermore, AMPK expression and phosphorylation are also significantly upregulated in WSSV-infected shrimp. Via the mTORC2-AKT pathway, AMPK phosphorylates glycolytic enzymes, promoting the expression of HIF-1α [111,112]. Thus, enhanced glycolysis provided energy and biomolecules for virus replication [110]. Additionally, Chen et al. [113] reported that an induced metabolic shift into the Warburg phenotype can counteract the high levels of ROS produced in response to WSSV infection.

**Table 2:** Mechanisms involved in the virus-induced shift towards aerobic glycolysis.

Virus	Mechanism	References
HCMV	Downregulation of GLUT1 Upregulation of GLUT4 and glycolytic intermediates. Increase in lactate production Involvement of AMPK pathway	[97,98,99,100,101]
SARS-CoV2	Induction and activation of HIF-1α Enhanced aerobic glycolysis	[82]
EBV	Upregulation of GLUT1, lactate dehydrogenase A, and PDK-1 LMP-1-induced expression of HIF-1α	[104,105,106]
KSHV	Induction and activation of HIF-1α Upregulation of GLUT3 and hexokinase II	[94]
NDV	Degradation of SIRT3	[108,109]
WSSV	Increased expression of hexokinase, phosphofructokinase, and AMPK Induction and activation of HIF-1α	[110,111,112]

GLUT: glucose transporter; AMPK: AMP-activated protein kinase; PDK-1: pyruvate dehydrogenase kinase 1; HIF-1α: hypoxia-inducible factor 1-alpha; LMP-1: latent membrane protein 1; SIRT3: sirtuin 3; HCMV: *human cytomegalovirus*; EBV: *Epstein–Barr virus;* KSHV*: Kaposi´s sarcoma-associated herpesvirus;* NDV: *Newcastle disease virus;* WSSV*: white spot syndrome virus.*

### 3.2. Reverse Warburg Effect

Oncoviruses can induce the reverse Warburg effect, which is characterized by enhanced aerobic glycolysis in cancer-associated fibroblasts. In this two-compartment model of metabolic symbiosis, cancer cells secrete H_2_O_2_ and cause oxidative stress in neighboring fibroblasts, which produce metabolic intermediates, such as pyruvate, ketone bodies, FA, and lactic acid that feed the tumor cells [114].

The life cycle of the oncovirus KSHV comprises a quiescent, latent state and a lytic, replicative phase. In contrast to the abundant expression of viral genes observed during lytic infection, latency is characterized by the episomic persistence of the viral genome with restricted viral gene expression [115]. The latency-associated nuclear antigen (LANA) protein is the main latency-regulatory viral protein, whereas the switch to the lytic phase is controlled by the replication and transcription activator (RTA) protein [116]. Different latency programs with distinct patterns of gene expression have been described. EBV nuclear antigen (EBNA) 1 is expressed in Burkitt’s lymphoma, LMPs in nasopharyngeal carcinoma, Hodgkin’s lymphoma, and NK/T cell lymphoproliferative diseases [117,118], and all EBNAs and LMPs are expressed in EBV-associated post-transplantation lymphoproliferative disorders, acute infectious mononucleosis, and X-linked lymphoproliferative syndrome [119]. It is well-established that in a latent KSHV infection, gene expression is restricted by several microRNAs (miRNAs) [120]. miRNAs are highly conserved noncoding RNAs that intervene in a wide array of biological processes [121]. Comparable to cellular miRNAs, virus-encoded miRNAs also regulate energetic metabolism and angiogenesis [122]. It has been demonstrated that KSHV-encoded miRNAs induce aerobic glycolysis in infected cells [123] while others are transferred to uninfected neighboring cells, inducing the reverse Warburg effect [79,124]. Induction of the glycolytic pathway is also observed in EBV-induced nasopharyngeal carcinoma. In this case, BART1-5P miRNAs increased glucose consumption and lactate production via regulation of the AMP-activated protein kinase (AMPK)/mTOR/HIF-1α pathway. The discovery of virus-encoded miRNAs unveiled a mechanism used by many viruses, particularly those that maintain a latent state, to induce a metabolic shift in the surrounding cells that favor their own persistence [125].

In general, viruses utilize a broad spectrum of strategies to directly or indirectly utilize the Warburg phenotype and mitochondrial metabolism to support the energetic and synthetic requirements of viral replication.

### 3.3. Fatty Acids Synthesis and Lipid Peroxidation

Disturbances in the synthesis, accumulation, and oxidation of FAs are common in viral infections. Lipid droplets (LDs), formed by a core of triacylglycerols, cholesteryl esters, and retinyl esters surrounded by a phospholipid monolayer, play an important role in viral infections [126]. Catabolism of stored triglycerides yields FAs that undergo mitochondrial β-oxidation, resulting in ATP formation. Aside from being used to store energy, FAs released from phospholipids and triglycerides act as signaling molecules [127,128,129]. During viral infections, changes in the lipid composition of LDs influence the pathogenicity and replication of the viruses [130]. For example, virus-dependent increased LD levels activate the NLRP3 inflammasome and modulate the innate immune response [131]. Below, we describe how viruses alter lipid metabolism.

**Figure 5:**
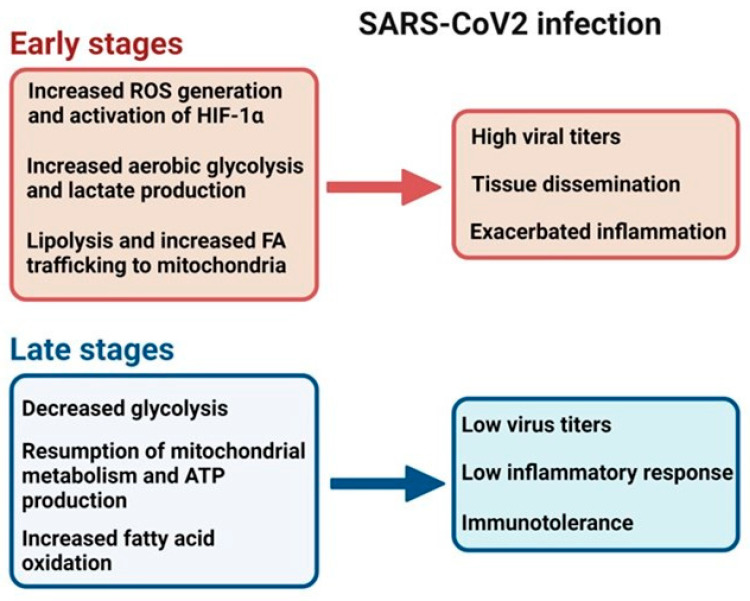
Metabolic switch in SARS-CoV2 infection. SARS-CoV2 infection follows a bimodal metabolic reprogramming. Initial SARS-CoV2 infection is characterized by mitochondrial ROS production that promotes hypoxia-inducible factor 1-alpha (HIF-1α) expression, lipolysis, and an increase in FA synthesis. Together with the induction of the Warburg effect, virus replication is enhanced, accompanied by a severe pro-inflammatory response (cytokine storm). During the second stage, glycolysis and oxygen consumption decrease, FA oxidation increases, and the mitochondria return to regular respiration and ATP production. It is a hypo-inflammatory stage, with decreased virus titers and immunotolerance [92,133].

Inhibition of FA synthesis decreases the production of infectious virus particles [132]. FA inhibition rendered KSHV intracellular virions non-infectious, suggesting a FA-dependent blockage of virion assembly and/or maturation [81]. By contrast, *gallid herpesvirus 2* or *Marek´s disease virus* (MDV), an alpha herpesvirus of chickens, increased FA synthesis in infected cells and induced LD formation, which facilitated virus replication [133]. Lipolysis has been described as a mechanism that provides energy for WSSV during virus replication. However, virus-induced lipogenesis is also required at later stages when long-chain FAs must be supplied for virus morphogenesis [134]. Recently, it was shown that the ORF6 protein of SARS-CoV2 that inserts into LDs and interacts with mitochondria binding to the SAM (β-barrel-specific sorting and assembly machinery) complex in the outer mitochondrial membrane induces lipolysis and stimulates FA trafficking into mitochondria [135,136]. Opposite to the initial stages of SARS-CoV2 infection, the second phase is characterized by low virus titers, reduced glycolysis, and oxidative metabolism, with increased FA oxidation [82]. The accessory SARS-CoV2 ORF3c protein, localized to the outer mitochondrial membrane, causes this metabolic shift, probably by an indirect mechanism that involves the transport of pyruvate from the cytoplasm to the mitochondrial matrix [136]. SARS-CoV2 infection demonstrates how a virus manipulates mitochondria according to the specific needs at each step of the infectious process (Table 3 and Figure 5).

Enveloped viruses need lipids for the viral envelope, while positive-sense RNA viruses require lipids to constitute the replication and assembly compartments [146]. These compartments are partially closed double-membrane structures formed by the rearrangement of membranes of different organelles. Infections with RNA viruses cause redistribution of LDs that become more accessible to viral non-structural proteins to initiate replication [147,148]. ROS generation also modulates LD formation. High mitochondrial ROS production increased the number of LDs and stimulated HBV gene expression in HepG2 human hepatocellular carcinoma cells expressing *hepatitis B virus* HBx protein [149]. Treatment with the antioxidant N-acetylcysteine decreased LD accumulation in a time-dependent manner [150]. In addition to the effect on LD formation, ROS induce the peroxidation of lipids rich in polyunsaturated FAs, both in cell membranes and viral envelopes. HCV infections cause liver steatosis and induce the peroxidation of lipids and viral enzymes of the replicase complex, particularly NS3/4A and NS5B [147]. Attenuated replication may facilitate long-term viral persistence [151]. It has been consistently demonstrated that peroxidation of the viral envelope causes disintegration of the viral particle [152]. The pathological role of lipid peroxidation has been demonstrated for neurotropic viruses, such as *rabies virus*. In a dorsal root ganglia model, Jackson et al. [153] demonstrated the presence of multiple axonal swellings, concomitantly with positive viral antigen and immunostaining for 4-hydroxynonenal (4-HNE), a marker of lipid peroxidation associated with oxidative stress. It was suggested that lipid peroxidation induced modifications in mitochondrial and cytoskeletal proteins, which were finally responsible for axonal swelling. Therefore, mitochondrial dysfunction and altered lipid metabolism, induced by virus replication, are key mediators of neuronal degeneration in *rabies virus* infection.

The cellular lipid metabolism in DENV-infected cells is characterized by activation of FA synthesis, accumulation of LDs, and mobilization of FAs [153], being FAs the main energetic substrate for efficient replication [154,155]. By an autophagy-dependent process, DENV increases β-oxidation, which generates ATP while depleting LDs and triglycerides [155].

Another alteration of lipid metabolism induced by viruses is caused by the immediate-early EBV protein BRLF1 that induces the expression of the FA synthase and several proteins involved in FA metabolism and cholesterol biosynthesis in epithelial and B cells [150].

Overall, alterations in lipid metabolism caused by viral infections range from increased FA synthesis and lipid droplet accumulation to increased FA oxidation and lipid peroxidation. Ultimately, the mechanisms seem to be virus-specific.

### 3.4. Glutamine Metabolism

Glutamine is the most abundant amino acid in the body and is used for the synthesis of proteins, nucleotides, and lipids [156]. Glutaminolysis is the catabolic conversion of glutamine into nitrogen-containing metabolites of the TCA cycle that both support OxPhos and are used for the synthesis of amino acids and nucleotides [157,158]. During viral infections, enhanced glutamine catabolism sustains virus replication [124].

The transcription factor *c-myc,* which regulates the expression of genes associated with cancer cell metabolism, favors viral infections [142]. The *adenovirus* E4ORF1 protein enhances adenovirus replication in human lung cells by activating c-*myc,* which, in turn, upregulates glutamine transporters and increases the activity of the rate-limiting enzyme glutaminase [159]. It has also been shown that during latency, the LANA protein of KSHV, as well as EBV infection, upregulates glutaminase expression by activating *c-myc* [160]. IAV infection of primary human bronchial epithelial cells and bone marrow-dendritic cells from pediatric patients showed a switch to glutamine utilization that was also dependent on *c-myc* [161].

Other viruses enhance glutaminolysis independently of *c-myc*. The highly virulent avian pathogen NDV induces upregulation of the glutamate transporter SLC1A3 and increases glutaminase activity [128]. In infected chicken embryonic fibroblasts with MDV, glutamine was converted to α-ketoglutarate as an intermediate for the TCA cycle to support virus replication [133]. Since glutamine is necessary for the efficient function of lymphocytes and macrophages [162], it was suggested that glutamine catabolism during MDV may contribute to avoiding the antiviral immune response [133]. A *murine norovirus* (MNV) model has been used to study metabolic reprogramming in MNV-infected macrophages. Noroviruses strains causing acute or persistent infection require glutaminolysis as a carbon source for genome replication. The viral non-structural protein NS1/2 has been identified as responsible for the increase in glutaminase levels. Although the effect of glutaminolysis on norovirus replication is clear, there is no current information on the effect of this catabolic process on infected macrophages [163]. Serum glutamine and glutamate depletion concomitantly with increased phosphatidylcholine biosynthesis suggest that both pathways contribute to HBV replication and progression to the hepatocellular carcinoma associated with HBV infection [164,165]. Glutaminolysis is also the major pathway supplying intermediates for the TCA cycle and OxPhos in HIV-1-infected naïve and memory CD4^+^ T cells [166,167]. Elevated levels of intracellular glutamine are observed during HIV-1 infection [167,168]. Although the entry of glutamine-derived carbon into the citric acid cycle is not affected, the secretion of glutamine-derived glutamic acid and protein levels of enzymes that metabolize glutamine to glutamic acid are significantly increased [168]. Glutaminolysis regulates the early steps of infection, favors virus replication, increases susceptibility to HIV-1 infection, and drives CD4^+^ T-cell proliferation [167,168].

Although many viruses require both increased glycolysis and glutaminolysis for efficient replication, the *vaccinia virus*, the causal agent of smallpox, only requires the anaplerotic reactions derived from glutamine catabolism without enhancing glycolysis. Glutamine is specifically used for *vaccinia virus* protein synthesis without a significant effect on transcription [169]. A similar finding has been described for the KSHV infection, in which glutaminolysis is required for early protein translation with no impact on virus gene transcription [79].

## 4. Mitochondria in the Immune Response to Viral Infections

The effects of ROS on the innate immune response are broad, altering protein function and introducing post-translational modifications that affect the immune signaling pathways. ROS participate in the antiviral immune response by inducing MAVS and by activating TLRs and DNA-sensing pathways, like STING (stimulator of interferon genes) [170,171]. A signaling cascade, initiated after nucleic acid-sensing receptors associated with the adaptor MAVS, leads to the expression of antiviral genes, mainly type I IFN. The robustness of the response depends on the activation/degradation of molecules like IRF-3 and IFN-stimulated genes (ISG) [172]. Other viruses have a direct effect on MAVS. The HCV protease NS3/4a binds to mitochondria and cleaves MAVS [173], whereas the PB1-F2 protein of IAV, as well as SARS-CoV2 ORF10 protein, induce mitophagy and MAVS degradation, suppressing the type I IFN response [174]. The *Murine herpesvirus-68* (MHV-68), closely related to KSHV and EBV, is also recognized by the cytosolic DNA-sensing pathway cGAS/STING. Tao et al. [170] demonstrated that increased ROS levels antagonized the IFN-β response to MHV-68 by oxidizing a STING-cysteine residue. Thus, by manipulating the ROS levels, MHV-68 inhibits the innate immune response, favoring its own replication.

Participation of ROS in the inflammatory response is also linked to their role as a second signal for activation of the NLRP3 inflammasome [172,175], which leads to cleavage and maturation of the pro-inflammatory cytokines, IL-1β and IL-18. Furthermore, Gasdermin-D, a key molecule during pyroptosis, is also cleaved upon ROS signaling [175]. Mitochondrial ATP, which is released after virus-induced cell death, has also been implicated in the activation of the NLRP3 inflammasome [175,176].

Mitochondrial DNA (mtDNA) is a double-stranded circular DNA that encodes 13 subunits of the mitochondrial respiratory chain. Its proximity to the inner mitochondrial membrane—where ROS are produced in the ETC—and the lack of protection by histones make mtDNA highly vulnerable. mtDNA plays a critical role in the mitochondrial-derived immune response [177]. Following mitochondrial damage or cell death, mtDNA is released and sensed by innate immune receptors, triggering the inflammatory response [3]. Several members of the *Picornaviridae* family, including *enterovirus 71* (EV-A71), *Seneca Valley virus* (SVV), and *foot-and-mouth disease virus* (FMDV), trigger mtDNA release into the cytosol after induction of mitochondrial damage. An opening of the mitochondrial permeability transition pore (mPTP) and VDAC1/Bak/BaX-dependent mtDNA leakage into the cytoplasm has been shown during SVV infection, whereas EV-A71 and FMDV also induce mPTP opening and VDAC1-dependent mtDNA release without involving Bak/BaX-activity. Released mtDNA also binds to cGAS (cyclic GMP-AMP synthase), activating the antiviral immune response. These picornaviruses are able to surpass the antiviral immunity by encoding the 2C protein, a highly conserved non-structural protein, which degrades cGAS or blocks the activation of the signaling cascade [178].

Independently of which is the mitochondrial target, viruses display an array of evasion strategies to avoid the mitochondria-derived immune response.

## 5. Virus Interaction with Voltage-Dependent Anion Channels

The voltage-dependent anion channels (VDACs) 1, 2, and 3 are β-barrel structures in the outer mitochondrial membrane of all eukaryotic cells [179,180,181,182]. The influx of oxidizable substrates, ADP, inorganic phosphate, and glycolytic ATP into mitochondria and the efflux of ATP through the outer mitochondrial membrane occurs only through VDACs [180,183,184]. VDACs also contribute to the regulation of calcium import to mitochondria [185]. Once inside the matrix, oxidizable substrates enter the TCA cycle, generating NADH that fuels the ETC. Overall, VDACs operate as a biological switch that, in the open state, maximizes the flux of metabolites for optimal mitochondrial function, whereas during the closed state, it lowers mitochondrial metabolism [183,186]. Thus, regulation of only this channel has an amplifying effect on several intra- and extra-mitochondrial pathways that modulate cancer metabolism and bioenergetics. Since mitochondrial ROS production directly depends on the activity of the ETC, VDAC opening or closing is a major driver for ROS formation [183]. Moreover, VDAC regulation may serve as an adjustable rheostat, with a range of operational levels that depend on the magnitude and duration of VDAC opening [183,186].

Information about the interactions between VDACs and viral proteins is still very scarce. The DENV E protein is a viral receptor binding protein that interacts with the cellular chaperone GRP78, which in turn interacts with VDAC1. As a result of these interactions, VDAC1 is re-localized during DENV infection, with mitochondria moving toward the endoplasmic reticulum. It was suggested that the re-localization of VDAC1 is required to traffic metabolites near the sites of DENV replication in proximity to the endoplasmic reticulum. The relevance of the DENV E/GRP78/VDACs was confirmed by VDAC1 silencing, which significantly reduced DENV protein expression, percentage of infection, and extracellular virus titers [187]. *Infectious bursal disease virus* (IBDV) is a double-stranded RNA (dsRNA) virus that causes severe immunosuppression in chickens. VDAC1 has been found to be upregulated during IBDV infection, being key in mediating IBDV polymerase activity [188]. VDAC1 interacts with IBDV VP1 and VP3 proteins, which are components of the polymerase complex. This interaction is crucial for stabilizing the complex and promoting polymerase activity that enhances viral replication and transcription. *Cytomegalovirus* (CMV) is a herpesvirus with high seropositivity in humans [189]. Although still controversial, it was suggested that CMV increases the risk of developing Alzheimer’s disease and cognitive alterations [190]. Recently, in a murine CMV model, an association between cognitive disorders and increased permeability of the blood–brain barrier was demonstrated. Among several altered mitochondrial parameters, it was shown that VDAC1 expression and mitochondrial ROS increased in brain microvascular endothelial cells after repeated MCMV infections during a 12-month period, which was suggestive of a cell dependence on OxPhos. Likely, cell metabolic reprogramming alters the integrity of the blood–brain barrier, favoring T-cell infiltration and facilitating cognitive decline [191].

The *enterovirus 71* (EV 71) 2B protein, which is a viroporin, directly interacts with VDAC3 to increase ROS production and enhance viral replication, as evidenced by the inhibition of EV 71 replication after VDAC3 knockdown. This interaction also suppresses the synthesis of taurine/hypotaurine, which has antioxidant activity. Although the exact mechanism is unknown, it was suggested that the 2B/VDAC3 interaction would suppress the expression of the enzymes involved in hypotaurine metabolism. By contrast, siRNA silencing of VDAC3 increased the antioxidant capacity of infected cells, implying that VDAC3 might be involved in a negative regulation of this antioxidant mechanism [192]. Ca^2+^ storage organelles, such as the ER and mitochondria, are important in calcium homeostasis [193]. Tight contact between mitochondria and ER triggers a rapid mitochondrial Ca^2+^ influx and Ca^2+^ overload that can disrupt Δψm and alter OxPhos [185]. Additionally, elevated mitochondrial Ca^2+^ levels increase ROS production [194,195] by stimulating the flow of electrons in the ETC or by altering the structure of the respiratory complexes [173]. VDACs participate in efficient Ca^2+^ transfer to the mitochondria by forming multi-protein complexes with Ca^2+^ channels in other organelles. The mitochondrial permeability transition pore (mPTP) is a non-specific pore that is permeable to solutes of <1.5 kDa. mPTP opens in the inner mitochondrial membrane under elevated levels of Ca^2+^ in the matrix, especially under conditions of oxidative stress and low levels of adenine nucleotides. The opening of the mPTP causes mitochondrial swelling, disruption of the outer membrane, and release of mitochondrial components that induce apoptosis. HCV core and NS5a proteins are known to cause ER stress with the release of Ca^2+^ and direct transfer from the ER to the mitochondria. This results in alterations of the ETC, increasing ROS production and sensitivity to mitochondrial permeability transition and cell death [196,197]. Particularly, the induction of mPTP has been attributed to HCV core protein [197]. HBV is another virus that modifies mitochondrial function by interacting with VDAC3. The HBV HBx regulatory protein has a binding affinity for VDAC3. The HBx/VDAC3 association decreased ΔΨ_m_ and altered mitochondrial physiology [198]. It is well-established that HBV can alter Ca^2+^ signaling to create a cellular environment favorable for virus replication [199]. Interaction of the viral protein with VDAC3 enhances Ca^2+ ^trafficking into the mitochondria. Furthermore, HBx regulates the mPTP, promoting mitochondrial Ca^2+ ^outflow [200]. Mitochondrial dysfunction during HBV infection has been linked to chronic hepatitis, cirrhosis, and oncogenesis. It is likely that the HBx/VDAC3 interaction and mPTP regulation mediated by HBx plays a pivotal role in the outcome of the disease. Pharmaceutical targeting of Ca^2+ ^signaling may provide a potent strategy for controlling HBV.

The *porcine respiratory and reproductive syndrome virus* (PRRSV) causes one of the most important diseases that affect the swine industry. PRRSV GP5 proteins are key for viral infectivity. Interaction of GP5 with VDAC1 promoted VDAC oligomerization and enhanced mitochondrial Ca^2+^ uptake from the ER by promoting ER-mitochondria contact. This resulted in the induction of mROS release and triggered autophagy, which repressed NLRP3 inflammasome activation and increased viral replication [201].

Although few, these studies demonstrate that the virus’s influence on VDAC function may be key to altering the physiological status of the mitochondria to sustain virus infection.

## 6. Viral Effects on the Electron Transport Chain

Several viruses have been shown to regulate the activity of ETC. RSV manipulation of the mitochondrial metabolism and signaling pathways has been widely recognized. Among several RSV-mitochondrial targets, the inhibition of Complex I is central to RSV pathogenesis [34]. High viral loads in infected cells are associated with decreased mitochondrial respiration and increased ROS production caused by the inhibition of complex I. It has been shown that HCV limits OxPhos by downregulating the core subunits of Complexes I and IV at early time points after infection, while Complex V activity decreases at the later stages [106]. Derakhshan et al. [202] demonstrated that the HSV-1 Us3 protein kinase mediated the inhibition of cellular respiration by blocking electron transport between complexes II and III. HIV-1 causes neurological disorders without infecting neurons. The neurotoxic HIV-1 transactivator of transcription (Tat) protein is secreted by infected T cells and macrophages/microglia affecting bystander cells, such as neurons [203]. Tat inserts into mitochondria through a basic domain, induces mitochondrial hyperpolarization, and decreases the activity of complexes III and IV in isolated neuronal mitochondria [204,205]. Thus, the effect of Tat on respiratory chain complexes in neurons and the global effects on mitochondrial function might be partially responsible for the neurological signs observed in HIV-1-infected patients. In the case of human *herpes simplex virus 1* (HSV-1) infection, a decline in ATP levels has been attributed to mitochondrial dysfunction [206]. The highly oncogenic MDV infects immune cells, causing a deadly lymphoproliferative disease in chickens [207]. The phosphorylated p38 protein (pp38) of MDV is required to lyse lymphocytes B, to induce latency, and to prevent apoptosis in T cells. pp38 increased the activity of mitochondrial succinate dehydrogenase, which is part of Complex II and feeds electrons directly into the ubiquinone/ubiquinol pool. The mechanism by which pp38 upregulates Complex II activity is currently unknown. However, co-localization with mitochondria could not be demonstrated, suggesting that the effect is indirect [208].

## 7. Virus-Triggered Mitochondrial Fission

Mitochondria are dynamic organelles that undergo morphological adaptations through cycles of fusion and fission to fuse or divide individual mitochondria, respectively [3]. Mitochondrial dynamics is involved in the regulation of the cell cycle, the immune response, and programmed cell death [209]. Beyond the changes in morphology, the number of mitochondria is determined by the rate of mitochondrial biogenesis and the removal of damaged mitochondria by mitophagy [210]. Disruption of mitochondrial fission leads to altered metabolism, proliferation, and apoptosis [211]. Some viruses exert a tight control on mitochondria dynamics. Checking when mitochondria fragmentation and mitophagy are required is clued to promptly escape from the mitochondria-induced immune response. AMPK is an energy sensor that detects low ATP levels [212,213] and affects mitochondrial dynamics. During NDV infection, under energetic stress, AMPK induces mitochondrial fission and controls mitophagy [108]. EBV-encoded BHRF1 protein is a BCL2 homolog that stimulates DNM1L (dynamin 1-like proteins)/Drp1-mediated mitochondrial fission and drives the reorganization of mitochondria into perinuclear aggregates. BHRF1 inhibits the IFN type I response by interacting with autophagosomes and stimulating mitophagy [214].

## 8. Concluding Remarks

Viruses modulate mitochondrial function and cellular metabolism to favor their own replication, persist in the host, and increase their virulence. Many viruses target more than one mitochondrial component and signaling pathway. Inhibition of the ETC, increased or decreased ROS production, differential utilization of glucose, glutamine, and FAs, and changes in mitochondrial dynamics are the mechanisms triggered by viral infections. Viruses use numerous strategies to bypass or avoid cellular mechanisms, which can prevent the infection from spreading or decrease the virulence. Several viruses are considered the main etiological agents of emerging diseases with pandemic potential. Therefore, besides understanding viral pathogenesis, gaining knowledge of virus/mitochondria interactions may provide novel opportunities for therapeutic interventions that can eventually be applied to the treatment of emergent viral diseases or to overcome the antiviral resistance of certain strains.

## Figures and Tables

**Table 3:** SARS-CoV2 accessory proteins and mitochondrial dysfunction.

Protein	Mitochondrial Target	Mitochondrial Dysfunction	References
ORF3a	mPTP	Increase mitochondrial Ca^++^ Increase ROS Promotes HIF-1α expression Release of mtDNA	[137]
ORF3b	MAVS-Drp1	Suppression of IFN response	[138]
ORF3c	TOM20 and TOM70 (OMM) MAVS	Increase of FA synthesis Suppression of IFN response	[136,139]
ORF7a	MAVS	Suppression of IFN response	[140]
ORF8a	MAVS	Suppression of IFN response	[141]
ORF9b	MAVS TOM70	Suppression of IFN response	[140,141,142,143,144]
ORF10	MAVS Mitophagy receptor NIX	Suppression of IFN response	[145]

mPTP: mitochondrial permeability transition pore; MAVS: mitochondrial antiviral-signaling protein; Drp1: Dynamin-related protein 1; LD: lipid droplet; SAM complex: Sorting and Assembly Machinery complex; OMM: outer mitochondrial membrane; TOM70: translocase of OMM.
